# Rationale, design and methods for the RIGHT Track Health Study: pathways from childhood self-regulation to cardiovascular risk in adolescence

**DOI:** 10.1186/s12889-016-3133-7

**Published:** 2016-06-01

**Authors:** Laurie Wideman, Susan D. Calkins, James A. Janssen, Cheryl A. Lovelady, Jessica M. Dollar, Susan P. Keane, Eliana M. Perrin, Lilly Shanahan

**Affiliations:** Department of Kinesiology, University of North Carolina Greensboro, Greensboro, 27402 NC USA; Department of Human Development and Family Studies, University of North Carolina Greensboro, Greensboro, 27402 NC USA; Department of Nutrition, University of North Carolina Greensboro, Greensboro, 27402 NC USA; Department of Psychology, University of North Carolina Greensboro, Greensboro, 27402 NC USA; Division of General Pediatrics and Adolescent Medicine, Department of Pediatrics, University of North Carolina Chapel Hill, Chapel Hill, 27599-7225 NC USA; Department of Psychology and Neuroscience, University of North Carolina Chapel Hill, Chapel Hill, 27599-3270 NC USA

**Keywords:** Longitudinal cohort, Childhood self-regulation, Cardiovascular risk factors, Adolescent health behaviors, Diet, Physical activity

## Abstract

**Background:**

Cardiovascular risk factors during adolescence—including obesity, elevated lipids, altered glucose metabolism, hypertension, and elevated low-grade inflammation—is cause for serious concern and potentially impacts subsequent morbidity and mortality. Despite the importance of these cardiovascular risk factors, very little is known about their developmental origins in childhood. In addition, since adolescence is a time when individuals are navigating major life changes and gaining increasing autonomy from their parents or parental figures, it is a period when control over their own health behaviors (e.g. drug use, sleep, nutrition) also increases. The primary aim of this paper is to describe the rationale, design and methods for the RIGHT Track Health Study. This study examines self-regulation as a key factor in the development of cardiovascular risk, and further explores health behaviors as an explanatory mechanism of this association. We also examine potential moderators (e.g. psychosocial adversities such as harsh parenting) of this association.

**Method/design:**

RIGHT Track is a longitudinal study that investigates social and emotional development. The RIGHT Track Health Study prospectively follows participants from age 2 through young adulthood in an effort to understand how self-regulatory behavior throughout childhood alters the trajectories of various cardiovascular risk factors during late adolescence via health behaviors. Individuals from RIGHT Track were re-contacted and invited to participate in adolescent data collection (~16.5, 17.5 and 18^+^ years old). Individuals completed assessments of body composition, anthropometric indicators, fitness testing (via peak oxygen consumption), heart rate variability during orthostatic challenge, 7-day accelerometry for physical activity and sleep, 24-h dietary recalls, and blood analysis for biomarkers related to metabolic syndrome, inflammatory status and various hormones and cytokines. Individuals also completed extensive self-report measures on diet and eating regulation, physical activity and sedentary behaviors, sleep, substance use, medical history, medication use and a laboratory-day checklist, which chronicled previous day activities and menstrual information for female participants.

**Discussion:**

Insights emerging from this analysis can help researchers and public health policy administrators target intervention efforts in early childhood, when preventing chronic disease is most cost-effective and behavior is more malleable.

**Electronic supplementary material:**

The online version of this article (doi:10.1186/s12889-016-3133-7) contains supplementary material, which is available to authorized users.

## Background

Obesity is a key risk factor for cardiovascular disease (CVD), and while obesity rates have remained relatively stable in children and adolescents in the past few years [1, 2], the obesity and overweight prevalence in adolescents aged 12–19 is still quite high (17.3 % for obesity (BMI ≥ 95th percentile) [1], and 32.2 % for overweight and obesity combined (≥85th percentile) [1]). Overweight and obesity status among young people is often accompanied by increases in other traditional cardiovascular risk factors, such as elevated lipids, altered glucose metabolism, and hypertension [3–9]. Systematic elevations of these factors constitute the metabolic syndrome, which is a robust predictor of morbidity and mortality in later life [10–17]. Alarmingly, estimated rates of metabolic syndrome in adolescents have more than doubled from 4.2 % in the early 90’s to 10.1 % in a more recent study [18]. Although it is not as extensively studied, elevated systemic low-grade inflammation is a correlate of obesity even at young ages [19]. Indeed, it is an independent marker of cardiovascular and metabolic disease risk [10–17, 19–21] and predicts adult CVD independent of metabolic syndrome [22–24]. Such elevated inflammation is relatively stable [25–27] and associated with vascular changes involved in atherosclerosis, which appear to be reversible during the early life course [28–32]. Thus, cardiovascular risk factors during adolescence—including obesity, elevated lipids, altered glucose metabolism, hypertension, and elevated low-grade inflammation—is cause for serious concern. Cardiovascular risk factors initiate and contribute to chronic disease processes and robustly predict subsequent morbidity and mortality by young adulthood or even earlier [33–38]. Despite the importance of these cardiovascular risk factors to health, most are rarely studied during adolescence, and very little is known about their developmental origins in childhood.

Although most research has focused on cardiovascular risk factors beginning in midlife or later, adolescence is emerging as a key period for potential increases in cardiovascular risk. Recent data from representative samples indicate that rates of several cardiovascular risk factors (e.g., hypertension, obesity, elevated low-grade inflammation) rise during adolescence*,* reaching relatively high levels by young adulthood [12, 18, 25, 39–41], and self-report of perceived health also declines beginning in mid-adolescence [42, 43]. In response to these trends, research studies focusing on prevention and intervention have examined and attempted to mitigate several early sources of cardiovascular risk factors; including “obesogenic” environments (neighborhood, home, childcare, school), media influences and other cultural and community factors as well as family factors, such as parental knowledge, attitudes, and modeling [44–47]. This work has contributed to immense progress in this field, and, quite possibly to the leveling off or even declining rates of pediatric obesity in the past 10 years [48–51]. Nevertheless, the prevalence of obesity remains high, offering an enduring impetus for more efficient translation of research-based interventions into professional practice guidelines that reduce obesity.

Research-based interventions with high levels of control for multiple variables are often expensive and burdensome for participants, limiting their utility for large scale dissemination. Ground-breaking new research suggests that deficits in self-regulation (sometimes also referred to as self-control) during childhood could be key to predicting adult disease risk, even when accounting for other risk factors such as poverty or low IQ [52]. Yet virtually no research to date has investigated whether this childhood individual-level risk factor is associated with increases in cardiovascular risk during adolescence and via which mechanisms. Adult chronic disease is concentrated among people who exhibited childhood deficits in self-regulation (SR) [52]. We use the term SR to refer to a specific set of processes or control mechanisms that function at the biological, emotional and behavioral level and enable individuals to manage arousal, attention, emotion, behavior, and cognition in adaptive ways [53–55]. Based on research among adults, both SR and cardiovascular risk factors appear to be associated with health behaviors (e.g., substance use, exercise, nutrition, and sleep). Given that SR emerges early and undergoes substantial development across childhood and into adolescence, an understanding of childhood emotional, behavioral, and physiological precursors to adolescent cardiovascular risk factors would inform and fine-tune prevention and intervention efforts that are aimed at disrupting pathways to adolescent cardiovascular risk, and ultimately at reducing the likelihood of adult CVD. This prospective-longitudinal study tracks changes in cardiovascular risk factors during late adolescence, a time during which parental control decreases dramatically, and SR may become more influential with respect to an individual’s health behaviors.

**Fig. 1:**
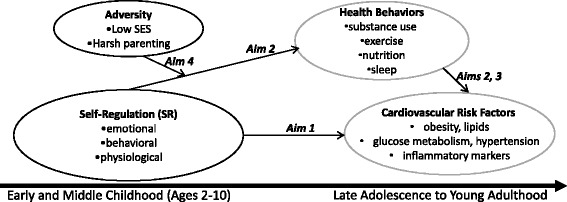
The potential pathways from childhood self-regulation to adolescent health behaviors and cardiovascular risk tested in the RIGHT Track Health Study. Childhood SR → health behaviors → adolescent CVR. Grey circles represent new data collection. Black circles represent historic data that are already collected

The ability of early SR to predict long-term health outcomes raised new hypotheses (grouped under four larger overall aims stated below) that are tested in the current study (see Fig. 1). To date, a fine-grained study of childhood SR, health behaviors and adolescent cardiovascular risk factors has not been possible due to a lack of extensive longitudinal data and excellent measures of these constructs. The current study utilizes an existing cohort studied from age 2, with extensive, state-of-the-art SR and psychosocial adversity data and collects diverse indicators of cardiovascular risk (traditional and non-traditional), and health behaviors in late adolescence. The specific aims of the study are; 1) to examine pathways from childhood SR to adolescent cardiovascular risk factors; 2) to test whether health behaviors mediate the childhood SR to adolescent cardiovascular risk factor pathways; 3) to establish directionality between health behaviors and cardiovascular risk factors and among indicators of cardiovascular risk during adolescence; and; 4) to test whether early SR to adolescent cardiovascular risk associations are moderated by the presence or absence of additional vulnerability factors, such as psychosocial adversity (e.g. poverty, harsh parenting).

## Methods/design

All procedures described in this manuscript have been approved by the University of North Carolina Greensboro Institutional Review Board (#11-0360; PI Wideman and #09-0427; PI Calkins) and the University of North Carolina Chapel Hill Institutional Review Board (#12-1817; PI: Shanahan). Pilot testing started in Fall 2012 and data collection for the first assessments with the current study protocol began in Spring 2014.

### Sample description

**Fig. 2:**
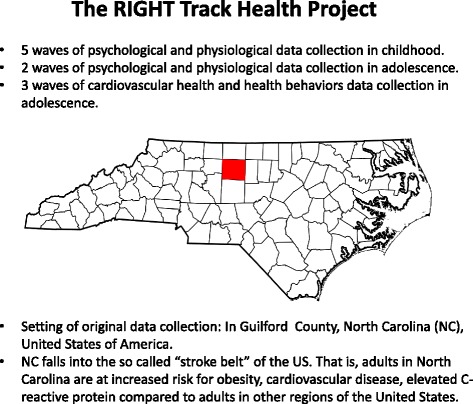
The geographical setting for the RIGHT Track Health Study

The current study recruits adolescents from three cohorts of individuals who are part of the ongoing, longitudinal study known as RIGHT Track, which investigates social and emotional development. The goal of the original recruitment was to obtain a sample of children who were at risk for developing future externalizing behavior problems and who were representative of the surrounding community in terms of race and socioeconomic status (SES). All cohorts were recruited through child day care centers, the County Health Department, and the local Women, Infants, and Children (WIC) program in central North Carolina (see Fig. 2 for setting). Potential participants for cohorts 1 and 2 were recruited at 2-years of age (cohort 1: 1994-1996 and cohort 2: 2000-2001) and screened using the Child Behavior Checklist (CBCL 2-3) [56], completed by the mother, in order to over-sample for externalizing behavior problems. Children were identified as being at risk for future externalizing behaviors if they received an externalizing T-score of 60 or above. Efforts were made to obtain approximately equal numbers of males and females. This recruitment effort resulted in a total of 307 children. Cohort 3 was initially recruited when infants were 6 months of age (in 1998) for their level of frustration, based on laboratory observation and parent report, and were followed through the toddler period (see Calkins et al. 2002 [57], for more information). Children from Cohort 3 whose mothers completed the CBCL at 2-years of age (*N* = 140) were then recruited for the larger study. Of the entire sample (*N* = 447), 37 % of children were identified as being at risk for future externalizing problems. There were no significant demographic differences between cohorts with regard to gender, χ^2^(2, *N* = 447) = .63, *p* = .73, race, χ^2^(2, *N* = 447) = 1.13, *p* = .57, or 2-year SES, *F* (2, 444) = .53, *p* = .59.

**Fig. 3:**
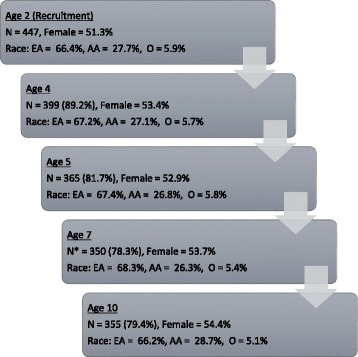
Sample attrition summary for childhood self-regulation assessments. Unless otherwise noted, there were no significant differences between those who participated at each time point compared to those who did not. As in most longitudinal studies, some participants were involved intermittently, participating at some assessment points, missing others, but then resuming their participation in the study at later time points. * denotes that families with lower 2 years SES were less likely to participate. EA = European American, A = African American, O = Biracial or Other Race

Of the 447 originally selected participants, six were dropped because they did not participate in any data collection at 2 years old. An additional 12 families participated at recruitment, did not participate at the 2-year assessment, but did participate at later years. At 4 years of age, 399 families participated. Families lost to attrition included those who could not be located, moved out of the area, declined participation, or did not respond to phone and letter requests to participate. At age 5 years, 365 families participated, including four who did not participate in the 4-year assessment and at 7 years of age, 350 families participated, including 19 who did not participate in the 5-year assessment. Families with lower 2-year SES, *t* (432) = -2.61, *p* < .01, were less likely to participate in the 7-year assessment. At age 10, 357 families participated, including 31 families who did not participate in the 7-year assessment. Independent of previous participation, attempts were made to recruit all families at every time point. Figure 3 provides detailed participation information at each age and statistical comparisons for participants versus non-participants based on key demographic variables.

### Historic data utilized in the current study

Historic data that were collected as part of the original RIGHT Track longitudinal study [NIMH 55625, NIMH 55584 & NIMH 58144] will be utilized as predictors in the current study to answer our specific aims. Measures of childhood adversity were collected at various time points throughout childhood (Table 1) and will be used to assess the role of vulnerability factors in predicting cardiovascular risk in adolescence. The following section includes a brief description and key references for other historic variables that will be used in the current study. For a detailed list of manuscripts that have utilized this historic data, please see Additional file 1.

**Table 1:** Examples of different historic measures of childhood adversity collected as part of the RIGHT Track study, measures at ages 2, 4, 5, 7, and 10 years old

Constructs	Example Measures	Example Variable	Reporter	Resources
Low socioeconomic status	Parental education and employment	Hollingshead index	Mother and father	Hollingshead (1975)
	Physical aggression, verbal aggression, discipline strategies, negative control, punitive reactions		Mother, father, observed	
Harsh parenting	Conflict Tactics Scale, Iowa Family Interaction Rating Scales: Instrument, Alabama Parenting Questionnaire, Home Observation for Measurement of the Environment (HOME), Coping with Children’s Negative Emotions Scale	Physical aggression, verbal aggression, discipline strategies, negative control, physical control, punitive reactions	Mother, father, observed (in laboratory), observed (in home)	Bradley, & Caldwell (1979)
				Straus (1979)
				Melby & Conger (2001)
				Smith, Calkins, Keane, Anastopoulos &, Shelton. (2004)
				Fabes, Eisenberg, & Bernzweig (1990)
				Fabes, Poulin, Eisenberg, & Madden-Derdich (2002).

The primary construct of interest from the childhood data is self-regulation. Self-regulation refers to the ability to modulate arousal and behavior in the context of specific environmental demands and these control processes function at the physiological, attentional, emotional, behavioral, cognitive, and social level [55]. Table 2 provides examples of the measures of physiological, emotional, and behavioral regulation that were assessed from ages two through ten. We also provide a more detailed explanation of the assessments of these constructs in the text that follows.

**Table 2:** Example measures of childhood physiological, emotional, and behavioral regulation a ages 2, 4, 5, 7, and 10 years old

Construct	Example Measures	*Example Variables*	*Reporter*	*Resources*
Physiological regulation	Lab-TAB Emotion-eliciting Tasks (i.e., Transparent Box, Puzzle Box), Continuous Performace Test (CPT), Tower Of Hanoi	RSA, RSA change, mean heart period, mean heart rate	Observed	Goldsmith, Reilly, Lemery, Longley, & Prescott (1993)
				Goldsmith & Rothbart (2010)
				Eisenberg et al. (2001)
				Connors (1994)
				Simon (1975)
Emotional regulation	Child Behavior Questionnaire (CBQ), Emotion Regulation Checklist (ERC), Lab-TAB Emotion-eliciting Tasks (i.e., Transparent Box, Puzzle Box, toy play/barrier task, perfect circle task), Child Behavioral Checklist (CBCL), The Behavioral Assessment System for Children (BASC), Child Depression Inventory (CDI), Diagnostic Interview Schedule for Children (DISC)	Negative affectivity, emotion regulation, distraction, help-seeking, venting, anger, fear, soothability, self-comforting, management of negative affect, depressive symptoms, symptoms of anxiety	Mother and father, Teacher, Observed	Putnam & Rothbart (2006)
				Shields & Cicchetti (1997)
				See above for Lab-TAB citations
				Achenbach & Rescorla (2001)
				Reynolds & Kamphaus (2004)
				Kovacs (1992)
				Shaffer, Fisher, & Lucas (1997)
Behavioral regulation	Snack Delay, Stroop Task, CPT, CBQ, naturalistic interaction, AD/HD Rating Scale-IV, Disruptive Behaviors Rating Scale	impulsivity, (dis)inhibition, compliance with instructions, defiance, self-distraction, persistence, approach	Observed, Mother and father	Kochanska., Murray, & Coy (1997)
				DuPaul, Anastopoulos, Power, Murphy, & Barkley (1994)
				Anastopoulos (1992)
				See above for CPT, CBQ citations

Physiological Self-regulation; Investigations of physiological markers of self-regulation have underscored the importance of the parasympathetic nervous system for regulating cardiac output under conditions of challenge [58]. Specifically, the myelinated vagus nerve, originating in the brainstem nucleus ambiguus, provides input to the sinoatrial node of the heart, producing dynamic changes in cardiac activity that allow the organism to transition between sustaining metabolic processes and generating more complex responses to the environment [59]. Of particular interest has been measurement of vagal regulation of the heart (indexed by decreases in respiratory sinus arrhythmia (RSA)) when the organism is challenged. Decreases in RSA when the individual is challenged, or vagal withdrawal, is linked to behavioral processes that are regulatory in nature [57, 60].

Physiological regulation procedures. Baseline RSA and RSA change, or vagal withdrawal, were assessed during engagement in developmentally appropriate tasks at each age. Of particular interest to studies of self-regulation is the collection of children’s RSA in a baseline task and tasks that elicit frustration.

At the 2-, 4-, and 5-year visits, children watched a 5-min segment of a neutral video about a puppy that explores its neighborhood to obtain baseline/resting RSA. At ages 7 and 10, children sat quietly to obtain baseline RSA. Children also participated in an age appropriate frustration/challenge task at each time point (Laboratory Temperament Assessment Battery: Locomotor Version 2.0; [61]). At age 2, children were given a toy or cookies in a locked box that they could not open. After 2 min, the experimenter came back in the room and helped the child to get the toy or cookies. At age 4, children were instructed to draw perfect circles and were told repeatedly for 3.5 min that the circles were not perfect; positive feedback was provided on the last circle drawn. At age 5, children participated in a task where an experimenter was to divide candy between themselves and the child. The experimenter gave themselves more candy than the child and also took the child’s candy and ate it. At ages 7 and 10 years, children participated in both behavioral and cognitive frustration tasks. For example, children put together towers of increasing difficulty, depicted in a picture, and participated in a Stroop color-naming interference task where the child was asked to read words by noting the color the word was printed in and not the word. Children also completed a task at ages 7 and 10, in which the child was presented a puzzle in a large box that was to be completed without looking at it; children could cheat by lifting a cloth that covered the front of the box.

Respiratory Sinus Arrhythmia (RSA). To collect heart rate data, the experimenter placed three disposable pediatric electrodes in an inverted triangle on the child’s chest. The electrodes were connected to a preamplifier, the output of which was transmitted to a vagal tone monitor (Ages 2, 4, 5, 7, & 10 years (cohort 1): VTM-I, Delta Biometrics, Inc., Bethesda, MD; Age 10 cohorts 2 and 3: Biolog 399x; UFI; Morro Bay, CA) for R-wave detection. A data file containing the interbeat intervals (IBIs) for the period of collection was transferred to a laptop computer for later artifact editing (resulting from child movement) and analysis. At ages 2, 4, 5, 7, and 10 years (cohort 1) measures of children’s RSA were obtained by editing IBI files using MXEDIT software (Delta Biometrics, Bethesda, MD). Cardio Batch/Edit software (Brain-Body Center, University of Illinois at Chicago, Chicago) was used to edit files for the 10 year visit for cohorts 2 and 3. Both software programs used the Porges (1985) [62] method of analyzing IBI data to calculate RSA. This method applies an algorithm to the sequential heart period (HP) data. The algorithm uses a moving 21-point polynomial to detrend periodicities in heart period that are slower than RSA. Then, a bandpass filter extracts variance in HP within the frequency band of spontaneous respiration, 0.24–1.04 Hz (ages 2, 4, 5, and 7) or 0.12–1.00 Hz (age 10). The natural log of this variance is taken and reported in units of ln (msec)^2^. To edit the files, the data were scanned for outlier points, relative to adjacent data. These outliers were replaced by dividing or summing them so that they would be more consistent with the surrounding data.

RSA was calculated every 30 s and the average across the 30-s epochs for each task is typically used in analyses. RSA change scores were computed by subtracting the frustration task RSA from baseline RSA [63–65]. Positive change scores reflect a decrease in RSA from baseline to the challenge task indicating RSA withdrawal.

#### Cardiovascular risk markers

Late adolescents report to the Exercise Physiology Laboratory at the University of North Carolina Greensboro at approximately ages 16.5 [also called Time 1 visit (T1)], 17.5 [also called Time 2 visit (T2)] and 18 or older [also called Young Adult visit (YA)] for assessment of various cardiovascular risk factors and health behaviors. The study design was chosen to minimize participant burden at the T2 visit, when significant data collection was still occurring for the original RIGHT Track longitudinal study on self-regulatory behaviors. Prior to arrival in the laboratory for the cardiovascular risk marker visit, participants are asked to abstain from vigorous exercise and alcohol consumption for the previous 24 h. In addition, all participants are asked to abstain from smoking the day of testing and to abstain from food for the 2 h prior to testing [66]. All participants are instructed to maintain adequate hydration in the 24 h prior to testing and water intake is encouraged prior to this testing session. Immediately prior to the start of the heart rate variability (HRV) data collection at the YA visit, participants are asked to void, since bladder distention has been shown to markedly alter sympathetic activity [67].

Upon arrival at the laboratory, all procedures for the specific visit (as outlined in Table 3), are explained in detail to the parent(s) (at T1 and T2 only) and to the adolescent. Consent and assent for the historic RIGHT Track assessments was provided for each set of testing procedures when the procedures occurred; this model of consent and assent is maintained at the T1 and T2 visits. At the YA visit, only participant consent is obtained.

**Table 3:** Assessment of cardiovascular risk markers and health behaviors in the RIGHT Track health study by time point of the study

Construct/Assessment	T1	*T2*	*YA*
*Cardivascular Risk Markers*			
BMI and overweight / obesity status	x	x	x
Anthropomorphic indicators (e.g., waist circumference, hip circumference, sagittal abdominal diameter )	x	x	x
Blood pressure and pre-/hypertension status	x	x	x
Serum markers of metabolic syndrome (e.g.,	x	x	x
Serum pro-inflammatory markers (e.g., C-reactive protein)	x	x	x
Serum anti-inflammatory markers	x	x	x
Body composition (e.g., body fat, fat mass, percent body fat)			x
Peak oxygen consumption			x
Heart rate variability during exercise and orthostatic challence			x
*Self-Reported Health Behaviors* (e.g., physical activity, eating behaviors, sleep, substance use)	x	x	x
*Objectively Assessed Health Behaviors*			
24-h Dietary Recalls (e.g.., Healthy Eating Index, total fruit and sodium intake, empty calories)	x		x
7-day Accelerometry (e.g., low intensity, moderate, vigorous physical activity; sedentary time; sleep patterns)			x

Biomarker Collection and Analyses; Adolescents are fasted from food, but allowed *ad libitum* water for at least 10 h prior to their visit. They are asked to limit their physical activity for this same time frame. To limit the influence of acute inflammation on our biomarkers, adolescents are immediately rescheduled if they report: 1) any illness in the past week or surgery in the past month; 2) immunizations within the past 2 weeks; or, 3) use of antibiotics, corticosteroids or other prescription anti-inflammatories within the past 10 days. Adolescents are semi-reclined (head and feet with similar elevation) in a blood draw chair. Whenever possible, blood is collected from an antecubital site, but when necessary, radial or hand venipuncture procedures are used. In all cases, blood is collected using pediatric gauge butterfly needles and a vacutainer system and universal precautions and OSHA guidelines are followed for blood handling. When necessary, pain reduction techniques such as use of the Buzzy® are employed to minimize patient anxiety. A total of 10 ml of blood is collected in serum separator tubes identified with only a subject ID and date. Blood is allowed to coagulate at room temperature for 20 min and spun at 4 °C for 15 min at 3000 rpm. To limit freeze/thaw cycling, serum is divided into multiple aliquots of 500-1000 μl and stored at -80 °C until analysis.

Inflammatory biomarkers and hormone levels in serum are analyzed by the Cytokine Analysis Facility at the University of North Carolina at Chapel Hill using the Multi-Plex (Bio-Rad, Hercules, CA) and the SpectraMax M2 ELISA plate reader. Samples are analyzed in duplicate with appropriate quality controls and all samples from a single individual are run in the same ELISA plate to minimize inter-assay variability and to insure comparability of assays across ages. Lipid profiles and glucose are assayed in the UNC Greensboro Exercise Physiology Laboratory using commercially available ELISA systems and the EPOCH plate reader (BioTek, Winooski, VT). Specifically, lipid profiles (total cholesterol, triglycerides, low-density lipoproteins and high-density lipoproteins) are assayed using colorimetric reagents (Wako USA, Richmond, VA) and glucose is assayed using a colorimetric assay (Caymen Chemical, Ann Arbor, MI). As outlined above for the inflammatory biomarkers, all samples from a given individual are assayed in the same ELISA plate.

Obesity Indices; At all data collection points, height is measured to the nearest 0.1 cm with a wall mounted, calibrated stadiometer (SECA, Chino CA) and weight is measured to the nearest 0.1 kg with a balance-beam scale (Detecto-medic, Brooklyn NY). Body mass index (BMI) is calculated using the standard formula [weight (kg)/height (m^2^)] and BMI percentiles were assigned by sex and age (in months) according to the most recent Center for Disease Control growth charts [68]. Healthy weight was defined as BMI >5th percentile and < 85th percentile for age and sex, overweight defined as values between the 85th and 95th percentile, and obesity defined by a BMI at the 95th percentile or greater [69]. Waist circumference (WC) and hip circumference (HC) are measured to the nearest 0.1 cm using a Gulick tension-tape measure by a sex-matched research assistant in a private location in the laboratory. WC is taken at the smallest part of the abdominal area (“natural waist”) and HC is taken at the maximal extension of the buttocks as outlined in the anthropometric standardization manual [70]. Both lying and standing sagittal abdominal diameter (SAD) are taken at the L4-L5 vertebral level to the nearest 0.1 cm using a Holtain-Kahn abdominal caliper (Croswell, UK).

At the YA visit only, the BODPOD (Cosmed, Concord, CA, USA), is used to assess body composition. The BODPOD is an air displacement plethysmography device that has been shown to be a valid and reliable tool for the measurement of body density across a range of individuals, including those who are overweight or obese, and is considerably less burdensome on participants than hydrostatic weighing [71, 72]. Standard manufacturer calibration procedures for volume are used with the scale calibrated daily using a 20 kg calibration weight. Participants enter the chamber wearing minimal clothing (spandex shorts, bra), and a swimming cap covering their hair where possible. Standard measurement procedures are followed. The subject’s thoracic lung volume is measured using the BODPOD breathing circuit system with calculations for body composition then performed using age and race appropriate algorithms built into the BODPOD system. The primary outcome variables are lean body mass, fat mass and percent body fat.

Blood Pressure; Upon arrival at the laboratory and after obtaining consent/assent, participants rest quietly for 5 min with arm supported at heart level and feet on the floor, as outlined by the Seventh Report of the Joint National Committee on Prevention, Detection, Evaluation and Treatment of High Blood Pressure [73]. Manual cuff blood pressure measurements are repeated two times with at least 5 min between measures. Resting heart rate is also taken twice at this time.

Peak Exercise Test (Fitness); A graded exercise test is performed in our study sample as a measure of cardiovascular fitness and to assess the heart rate and autonomic response to exercise at the YA visit only. Given the additional components of the exercise testing paradigm that are part of the visit (i.e. the HRV assessment), the duration of the exercise test has to be similar across participants. Yet, the duration of many traditional graded exercise tests is highly dependent on individual fitness level, making many of these inappropriate choices for the current study. The goal of the pilot testing was to find a protocol that would result in an individual attaining volitional fatigue in 10–12 min following the onset of exercise. Pilot testing determined that a Modified Treadmill Protocol should be used for our adolescent population.

The actual exercise testing protocol consists of an initial 3 min period with the goal of developing comfort with the treadmill and achieving a self-determined speed (walking or jogging) that one would anticipate being able to maintain for at least 10–15 min. At the end of the 3-min period, the treadmill speed remains constant for the remainder of the test, with the incline increasing by 3 % every 2 min after that point (0 % @ 3 min, 3 % @ 6 min, 6 % @ 9 min, and onwards) until volitional fatigue, or test termination by the research staff. Certified ACSM Clinical Exercise Physiologists are responsible for conducting the exercise testing. Participant health information is adjudicated by study staff with reference to the ACSM Guidelines for Exercise Testing and Prescription [74]. Because there is no medical supervision, some individuals may only be cleared for sub-maximal exercise testing in this environment. These participants will undergo the same testing procedures; however, the test is terminated when 85 % of the age-predicted maximal heart rate is reached.

Expired air is collected through a Hans Rudolph mouthpiece and gas analysis is performed using the Parvo Medics TrueOne 2400 (Parvo, USA). Standard gas and flow calibration procedures are completed prior to every test following manufacturer instructions. Five minutes of seated resting breathing prior to the test allows the participant to get accustomed to breathing with the mouthpiece. During the exercise test, heart rate is monitored real-time using the Polar V800 (Polar, Finland), and the Rating of Perceived Exertion (RPE), obtained at the end of each stage [75]. Peak oxygen consumption, defined as the highest value attained during testing, is the primary outcome for the exercise test. This measure is a robust indicator of overall health status and is a promising, yet rarely used marker of CVD risk in this population. Heart rate recovery dynamics are assessed by monitoring heart rate in a seated position for 10 min following the cessation of exercise. Participants who complete a sub-maximal versus peak exercise test will be treated separately in analyses that utilize the exercise data.

Heart Rate Variability; An orthostatic challenge is used to assess autonomic function, via changes in heart rate variability (HRV), to a mild physiologic stressor (postural change) while a peak exercise session is used to assess changes in autonomic function in response to a major physiologic stressor. These physical stressors are used as a means of comparing autonomic function to the psychological stressor paradigms that participants underwent during their early developmental years as part of RIGHT Track. While the measures of HRV collected during this session are not directly comparable to the measures of HRV collected during the early years of the RIGHT Track study, they will provide complementary information regarding the role of the autonomic nervous system as a regulator of cardiac control. Variability in heart rate (R-R intervals), is collected un-paced at rest using a wireless BioNomadix ECG with respiration strap connected to the BIOPAC MP150 (Biopac Systems, Goleta, CA, USA). Participants spend 5 min supine, 5 min seated and 5 min standing in a small, lit room with potential visual and auditory distractions minimized. While high frequency HRV may be recorded over periods as short as 60s [76], low frequency HRV requires long periods of data collection for reliable spectral information. In addition, 5 min of data collection in each postural position provides adequate data points to assess regularity metrics, such as approximate entropy. The Acknowledge software program (Biopac Systems, Goleta, CA, USA) collects the data real-time, and exported data is processed and analyzed for time, frequency and non-linear domains using Kubios software (University of Eastern Finland). Artifacts are removed using manual checks, and artifact correction and de-trending is completed with the Kubios software. The orthostatic challenge is completed both pre-exercise and approximately 20 min post exercise. During the exercise portion of the visit, heart rate and HRV are also assessed using a Polar RS800CX (Polar, Finland). Data files are downloaded using the Polar ProTrainer 5 software (Polar, Finland) and exported to Kubios for further cleaning, processing and analysis.

#### Health behaviors

Dietary Recall & Analyses; At T1 and YA, dietary recalls are assessed by the Nutrition Obesity Research Center (NORC) at the University of North Carolina Chapel Hill. Dietary intake data is collected and analyzed using the Nutrition Data System for Research (NDSR) software developed by the Nutrition Coordination Center (NCC), University of Minnesota, Minneapolis, MN. Three 24-h dietary recalls are collected by telephone (2 weekdays and one weekend day). To increase the accuracy of recall, a trained interviewer conducts the sessions using a multiple-pass procedure for recalling food intake [77]. First, a list of all foods and beverages consumed in the past 24 h is obtained from the participant. Next, this list is reviewed with the participant for completeness and accuracy. Then the amount consumed and the method of preparation of each item is collected. Finally, the detailed information is reviewed again with the participant for completeness and correctness. The recall is then reviewed by the NORC coordinator using the quality assurance guidelines of NDSR. This method has been validated against doubly-labeled water as an accurate measure of energy intake [78]. To reflect the marketplace throughout the study, dietary intake data is analyzed using multiple versions of the Nutrition Data System for Research software. Installation of software upgrades are based on the currently available version and timed so that data collection is not disrupted. Final calculation is completed using the most current NDSR version. The NDSR time-related database updates only analytic information and maintains nutrient profiles in the original version used for data collection.

Data from the 3 days of recall is averaged to determine the quality of the diet, using the Health Eating Index (HEI) 2010 score [79]. The HEI is based on the dietary recommendations of the 2010 Dietary Guidelines for Americans. It consists of 12 components: total fruit, whole fruit, total vegetables, greens and beans, whole grains, dairy total protein foods, seafood and plant proteins, fatty acids, refined grains, sodium, and empty calories to assess the quality of the diet. A score is assigned to each component and then summed (range of total score: 0 to 100). The HEI scores are calculated from the nutrient and food group outputs as described by Wiltheiss et al. [80]. Data from the NDSR is also used to assess energy and other nutrient intake.

Health Behavior Questionnaires; At all adolescent data collection time points (T1, T2 and YA), individuals complete several self-report measures of health behaviors (see Table 4 for details). In addition, at T2 only, participants complete a sports history questionnaire to collect information on sport engagement at younger ages and the primary reasons for continued engagement versus attrition from sport activity. The sports history questionnaire is currently being validated in our RIGHT Track Health sample. At T1 and T2 only, parents also complete modified items from the Behavioral Risk Factor Surveillance System (BRFSS) about their own diet and exercise behaviors.

**Table 4:** Key self-reported measures of adolescent health behaviors collected at adolescent time points (Time 1, Time 2 and Young Adult)

Measure	Subscales	Resources
*Diet and Eating Regulation*		
Three-Factor Eating Questionnaire	Restraint	Stunkard & Messick (1985)
	Disinhibition	
	Hunger	
Adolescent Food Habits Questionnaire	Adolescent healthy eating behavior (global summary scale indicative of consumption of specific foods, food purchases and preparations; items refer to both healthy & unhealthy behaviors.)	Johnson, Wardle, & Griffith (2002)
*Physical Activity and Sedentary Behaviors*		
Godin Leisure-Time Exercise Questionnaire	Light	Godin & Shephard (1985); Jacobs et al. (1993)
	Moderate	
	Strenuous	
	Total	
	“Sweat-Breaking” Physical Activity	
Sedentary Behavior	Total inactive hours	Gortmaker et al. 1999; Utter et al 2003; Harvard School of Public Health Nutrition Dept WebPage; http://regepi.bwh.harvard.edu/health
	Hours of TV/DVD/video viewing	
	Hours of computer use	
	Hours of sedentary electronic games	
*Sleep*		
Pittsburgh Sleep Quality Index	Sleep Quality	Buysse, Reynolds, Monk, Berman, & Kupfer (1988)
	Sleep Latency	
	Sleep Duration	
	Habitual Sleep Efficiency	
	Sleep Disturbance	
	Use of Sleep Medication	
	Daytime Dysfunction	
	Global Score	
*Instruments Assessing Multiple Health Behaviors*		
Youth Risk Behavior Survey	Tobacco Use	Brener, Collins, Kann, Warren, & Williams (1995); Centers for Disease Control and Prevention (2012)
	Alcohol & Other drug use	
	Dietary Behaviors	
	Physical Activity	

Accelerometry; Following the completion of the YA visit, participants are given an Actigraph GT9X Link accelerometer (Actigraph LLC, FL, USA) and instructed to wear it on their non-dominant wrist 24 h per day for the next 7 days. Participants are advised to remove the device for water-based activities, and where regulated by sporting competition or occupation. In addition to built-in wear time validation functions, participants also record non-wear time on a log sheet. After 7 days of wear, participants mail the device and log sheet back to the study team and compliance is assessed. Individuals who do not meet the minimal wear time criteria (wore the device less than 4 days), will be resent an accelerometer for a further wear period. More than 4 days of wear time is recommended for the assessment of usual physical activity, however it is currently unknown what the minimum wear period is when utilizing a 24 h protocol, so we decided to use conservative measures. It is suggested that 6 days are required for accurate assessment of sedentary time [81] and 4–7 days are required for accurate assessment of sleep patterns in children [82], but no wear time validation for sleep patterns is currently available for adolescents. Accelerometers are set up to collect at 30Hz for the 7 day period and downloaded into the Actilife software (Actigraph LL, FL, USA). The wrist worn protocol was selected to reflect current data collection procedures used by NHANES, and to allow the collection of objective measurement of sleep, which is a health behavior that is receiving increasing attention for its important role in physical and mental health in youth. Preliminary data from the NHANES switch to wrist worn accelerometry has identified significantly improved compliance over traditional waist worn procedures [83]. Additionally, the number of hours of wear time has been shown to influence estimates of physical activity, so a 24 h protocol may assist in ensuring the accuracy of measurement [84]. With several years of data collection required for the present study, and the advancing nature of techniques in the processing and analyses of accelerometry derived information, it is currently unknown which algorithms and methods will be used as our final outcomes. All accelerometry files will be batch processed at the end of the data collection utilizing currently accepted and validated standards. It is expected that time spent in sedentary (sedentary time), in low intensity physical activity (LPA) and in moderate-to-vigorous physical activity (MVPA) will be computed using established thresholds (sedentary time: <100 cpm, LPA: 100–2295 cpm, MVPA: ≥ 2296 cpm) [85, 86].

Control Variables and Potential Confounds: At each age, participants bring their prescribed medications to the laboratory; interviewers record dosage, frequency of use, and reason for use. Glucocorticoids, anti-inflammatory and immunosuppressant drugs are of special interest with respect to inflammation, although aggregate measures of any medication use tend to be robustly associated with inflammatory markers in adolescents [27]. We also administer a “Laboratory Day Checklist” which measures sleep, exercise, and substance use in the 24 h before the laboratory visit. Females are also asked to report the first day of their last menstrual period, information about their typical menstrual cycles, and use of hormonal contraceptives. We also assess family history of health, with a special focus on autoimmune, inflammatory, and cardiovascular health conditions. The laboratory day checklist is administered before the blood draw and closely assesses any medical conditions that would preclude us from conducting the draws (e.g., blood disorders). In addition to these control variables, a number of constructs that could be associated with self-regulation, health behaviors, and cardiovascular risk markers are assessed, including somatic symptoms with the Children’s Somatization Inventory (CSI-24) and depressive symptoms with the Short Mood and Feelings Questionnaire. In addition, the original ongoing longitudinal RIGHT Track study assessed a wealth of self-regulation and psychological variables during adolescence (i.e., ages 15.5 and 17.5) that can be included as control variables in our analyses.

## Discussion

The main aim of the RIGHT Track Health Study is to examine the role of childhood physiological, behavioral, and emotional self-regulation in the development of adolescent cardiovascular risk factors and to test the role of health behaviors in these associations. This current study leverages an ongoing 15^+^-year study with a prospective-longitudinal community study extending from childhood onwards and uses high-quality, repeated multi-method, biobehavioral measures of emotional, behavioral, and physiological SR and high-quality, repeated multi-method measures of psychosocial adversity. During adolescence, the RIGHT Track Health Study adds valid, longitudinal assessments of CVR and health behaviors. The current paper provides basic methods for collecting original/historic measures on this sample (starting at the age of 2 years) and detailed information on the cardiovascular risk and health behavior data assessed during adolescence and into young adulthood.

The fine-grained study of childhood self-regulation, health behaviors and adolescent cardiovascular risk has been limited by both a dearth of longitudinal data and a lack of extensive measures of these constructs. One of the strengths of the RIGHT Track Health Study is the inclusion of multiple data collection time points during childhood and an assessment of self-regulation in terms of physiological, emotional, and behavioral domains, which allows this study to test whether poor self-regulation between ages 2 to 10 manifests itself in increases in cardiovascular risk in late adolescence. Further, we can test whether specific domains of self-regulation (behavioral, emotional or physiological) play a unique role in cardiovascular risk or whether the accumulation of self-regulatory deficits across domains is more significant.

In addition, we will have multiple biomarker data collections within late adolescence and young adulthood, developmental periods when most individuals are navigating major life changes (e.g., changes in social and family relations and schooling) and gaining increasing autonomy from their primary caregivers. During this time, health behaviors likely increasingly reflect a young person’s own choices and become increasingly stable with age. Indeed, the childhood self-regulation → adolescent health behavior links that we examine here may be critical in long term cardiovascular and other health outcomes. Our data will allow us to test whether health behaviors in adolescence, including exercise, nutrition, sleep and substance use mediate linkages from self-regulation in childhood to cardiovascular risk in adolescence and to investigate the directionality of the link between health behaviors and cardiovascular risk during this developmental period.

An additional major strength of the current study is the longitudinal assessment of childhood adversity (i.e. poverty, harsh parenting). Psychosocial adversity is associated with both poor self-regulation and later cardiovascular risk [87–90]. Our study will test whether children with regulatory difficulties who are also exposed to childhood adversity have higher levels of cardiovascular risk. We will also explore patterns of this relationship for males and females and for European and African American individuals.

One limitation of the RIGHT Track Health Study is that our sample is limited to the individuals who were originally enrolled in the longitudinal study when they were 2 (approximately 1.5 decades ago). Over the years, attrition in the cohort due to geographic relocation of families and loss of contact with some participants limits our ability to follow every participant. In addition, as in most longitudinal studies, some individuals were only able to participate intermittently, missing some assessments, but resuming their participation in the study at later time points. Thus, we recognize that some bias may exist in our sample since participants who remained involved may have lower geographic mobility and/or intrinsic interests that motivate them to continue participating in health-related research. However, the RIGHT Track Study has found limited support for selective attrition and attrition rates for the study are similar to other high-quality longitudinal studies. Participants who continued versus those who ceased involvement did not significantly differ on many key characteristics, including socioeconomic status, race, and gender. Whether this pattern in attrition will hold in adolescence remains to be seen with our new data collection.

The RIGHT Track Health Study data will provide valuable information for youth health care and may inform policy makers about the specific health behaviors (e.g. diet, physical activity, sleep, substance use) that are most influenced by early childhood regulatory abilities. In addition, assessing the trajectories of these cardiovascular risk factors from childhood to adolescence may indicate critical ‘windows’ for reducing certain risk factors.

In summary, individuals in the RIGHT Track Health Study are prospectively followed from age 2 through young adulthood in an effort to understand how self-regulatory behavior throughout childhood alters the trajectories of various cardiovascular risk factors during late adolescence via health behaviors. Insights provided by these data could contribute to our understanding of chronic disease prevention early in life when intervention efforts are most cost-effective and behavior may be more malleable [91].

## Abbreviations

BMI, body mass index; CVD, cardiovascular disease; HC, hip circumference; HEI, healthy eating index; HP, heart period; HRV, heart rate variability; LPA, low intensity physical activity; MVPA, moderate-to-vigorous physical activity; NDSR, nutrition diet system for research; NORC, Nutrition Obesity Research Center; RPE, rating of perceived exertion; RSA, respiratory sinus arrhythmia; SAD, sagittal abdominal diameter; SES, socioeconomic status; SR, self regulation; T1, time 1; T2, time 2; WC, waist circumference; YA, young adult

## Additional file

Additional file 1: RT Empirical Publications List. (DOCX 22 kb)
