# Supplementary material for: Rationale, design and methods for the RIGHT Track Health Study: pathways from childhood self-regulation to cardiovascular risk in adolescence
Source: BMC Public Health. 2016 Jun 1;16:459. doi: 10.1186/s12889-016-3133-7 (PMC4888421; doi:10.1186/s12889-016-3133-7)
Supplement: Additional file 1: — RT Empirical Publications List. (DOCX 22 kb) [file 12889_2016_3133_MOESM1_ESM.docx]

Publications

*In Print*

Rose, M. J., Calkins, S. D., Keane, S.P., Shanahan, L., O’Brien, M. (2016). Relational aggression predicts perceived popularity in elementary school children. *Journal of Early Adolescence*. (in press)

Nelson, J. A., Perry, N. B., O’Brien, M., Calkins, S. D., Keane, S. P., & Shanahan, L. (2016). Mothers’ and fathers’ reports of their supportive responses to their children’s negative emotions over time. *Parenting: Science And Practice*,*16*(1), 56-62.

Blair, B. L., Perry, N. B., O’Brien, M., Calkins, S. D., Keane, S. P., & Shanahan, L. (2015). Identifying developmental cascades among differentiated dimensions of social competence and emotion regulation. *Developmental Psychology*, *51*(8), 1062-1073.

Mackler, J. S., Kelleher, R. T., Shanahan, L., Calkins, S. D., Keane, S. P., & O'Brien, M. (2015). Parenting stress, parental reactions, and externalizing behavior from ages 4 to 10.*Journal Of Marriage And Family*, *77*(2), 388-406.

Blair, B. L., Perry, N. B., O’Brien, M., Calkins, S. D., Keane, S. P., & Shanahan, L. (2014). The indirect effects of maternal emotion socialization on friendship quality in middle childhood. *Developmental Psychology*, *50*(2), 566-576.

Perry, N. B., Mackler, J. S., Calkins, S. D., & Keane, S. P. (2014). A transactional analysis of the relation between maternal sensitivity and child vagal regulation. *Developmental Psychology*, *50*(3), 784-793.

Morris, N., Keane, S., Calkins, S., Shanahan, L., & O'Brien, M. (2014). Differential components of reactivity and attentional control predicting externalizing behavior. *Journal Of Applied Developmental Psychology*, *35*(3), 121-127.

Shanahan, L., Calkins, S. D., Keane, S. P., Kelleher, R., & Suffness, R. (2014). Trajectories of internalizing symptoms across childhood: The roles of biological self-regulation and maternal psychopathology. *Development And Psychopathology*, *26*(4, Pt 2), 1353-1368.

Graziano, P., Kelleher, R., Calkins, S., Keane, S., & O’Brien, M. (2013). Predicting weight outcomes in preadolescence: The role of toddlers’ self-regulation skills and the temperament dimension of pleasure. *International Journal of Obesity, 37,* 937-942. PMCID: PMC3543516

Hinnant, J. B., Nelson, J. A., O'Brien, M., Keane, S. P., & Calkins, S. D. (2013). The interactive roles of parenting, emotion regulation and executive functioning in moral reasoning during middle childhood. *Cognition And Emotion*, *27*(8), 1460-1468.

Nelson, J. A., O'Brien, M., Calkins, S. D., & Keane, S. P. (2013). Mothers' and fathers' negative responsibility attributions and perceptions of children's problem behavior. *Personal Relationships*, *20*(4), 719-727.

Graziano, P., Calkins, S.D., Keane, S.P., O’Brien, M. (2011). Cardiovascular regulation profile predicts developmental trajectory of BMI and pediatric obesity. *Obesity, 19*, 1818-1825. PMID: 21546929

Graziano, P., Keane, S. P., & Calkins, S. D. (2011). Sustained attention development from toddlerhood topreschool period: Associations with toddlers’ emotion regulation strategies and maternal behavior. *Infant and Child Development. 20*(5), 389-408. PMID: 22121338

Waite, E. B., Shanahan, L., Calkins, S. D., Keane, S. P., & O'Brien, M. (2011). Life events, sibling warmth, and youths' adjustment. *Journal Of Marriage And Family*, *73*(5), 902-912.

**Blandon, A. Y., Calkins, S. D., Grimm, K. J., Keane, S. P., & O'Brien, M. (2010). Testing a developmental cascade model of emotional and social competence and early peer acceptance.*Development and Psychopathology. Special Issue: Developmental Cascades: Part 2, 22*, 737-748. PMCID: PMC3019307**

Blandon, A. Y., Calkins, S. D., Keane, S. P., & O’Brien, M. (2010). Contributions of child’s physiology and maternal behavior to children’s trajectories of temperamental reactivity. *Developmental Psychology, 46*(5), 1089-1102. PMCID: PMC3035931

Blandon, A. Y., Calkins, S. D., & Keane, S. P. (2010). Predicting emotional and social competence during early childhood from toddler risk and maternal behavior. *Development and Psychopathology, 22*(1) 119-132. PMCID: PMC2860175

Graziano, P., Calkins, S. D., & Keane, S. P. (2010). Maternal behavior and children’s early emotion regulation skills differentially predict development of children’s reactive control and later effortful control. *Infant and Child Development, 19(4),* 333-353. PMCID: PMC3034150

Graziano, P., Calkins, S. D., & Keane, S. P. (2010). Toddler self-regulation skills predict risk for pediatric obesity. *Journal of International Obesity, 34,* 633-641*.* PMCID: PMC2854309

Mokrova, I., O’Brien, M., Calkins, S. D., & Keane, S. P. (2010). Parental ADHD symptomology and ineffective parenting: The connecting link of home chaos. *Parenting: Science and Practice.* PMCID: PMC2864040

Reavis, R., Keane, S. P., & Calkins, S. D. (2010). Trajectories of peer victimization: The role of multiple relationships. *Merrill-Palmer Quarterly*. PMCID: PMC2951683

Topor, D.R., Keane, S.P., Shelton, T.L., & Calkins, S.D. (2010). Parent involvement and student academic performance: A multiple meditational analysis. Journal of Prevention & Intervention in the Community, 38(3), 183-197. PMCID: PMC3020099

**Calkins, S. D., & Keane, S. P. (2009). Developmental origins of early antisocial behavior. *Development & Psychopathology, 21*(4), 1095-1109. PMCID: PMC2782636**

Nelson, J., O’Brien, M., Blankson, N., Calkins, S. D., & Keane, S. P. (2009). Family stress and parental responses to children's negative emotions: Tests of the spillover, crossover, and compensatory hypotheses. *Journal of Family Psychology, 23*(5), 671-679. PMCID: PMC2855124

Berdan, L. E., Keane, S. P., & Calkins, S. D. (2008). Temperament and externalizing behavior: Social preference and perceived acceptance as protective factors. *Developmental Psychology, 44*, 957-968*.* PMCID: PMC2773664

Blandon, A. Y., Calkins, S. D., Keane, S. P., & O’Brien, M. (2008). Individual differences in trajectories of emotion regulation processes: The effects of maternal depressive symptomatology and children’s physiological regulation. *Developmental Psychology, 44*, 1110-1123. PMCID: PMC2630713

Calkins, S. D., Graziano, P. A., Berdan, L. E., Keane, S. P., & Degnan, K. A. (2008). Predicting cardiac vagal regulation in early childhood from maternal-child relationship quality during toddlerhood. *Developmental Psychobiology, 50*, 751-766. PMCID: PMC 2860183

Degnan, K. A., Calkins, S. D., Keane, S. P., & Hill, A. L. (2008). Profiles of disruptive behavior across early childhood: Contributions of frustration reactivity, physiological regulation and maternal behavior. *Child Development, 79*, 1357-1376. PMCID: PMC2823572

**Calkins, S. D., Blandon, A. Y., Williford, A. P., & Keane, S. P. (2007). Biological, behavioral and relational levels of resilience in the context of risk for early childhood behavior problems. *Development & Psychopathology, 19*, 675-700*.***

**Calkins, S. D., Graziano, P., & Keane, S. P. (2007). Cardiac vagal regulation differentiates among children at risk for behavior problems. Biological Psychology*, 74*, 144-153. PMCID: PMC2773670**

Graziano, P., Keane, S. P., & Calkins, S. D. (2007). Cardiac vagal regulation and early peer status. *Child Development, 78*, 264-278.

Graziano, P., Reavis, R., Keane, S. P., & Calkins, S. D. (2007). The role of emotion regulation in children’s early academic success. *Journal of School Psychology, 45*, 3-19.

Williford, A. P., Calkins, S. D., & Keane, S. P. (2007). Predicting change in parenting stress across early childhood: Child and maternal factors*.* *Journal of Abnormal Child Psychology, 35*, 251-263*.*

Hill, A. L., Degnan, K. A., Calkins, S. D., & Keane, S. P. (2006). Profiles of externalizing problem behavior across preschool: The role of emotion regulation and inattention. *Developmental Psychology, 42*, 913-928.

Smith, C. L., Calkins, S. D., & Keane, S. P. (2006). The relation of maternal behavior and attachment security to toddlers’ emotions and emotion regulation. *Research in Human Development, 3*, 21-31.

Calkins, S.D., Hungerford, A., & Dedmon, S.E. (2004). Mothers’ interactions with temperamentally frustrated infants. *Infant Mental Health Journal, 25,* 219-239.

**Calkins, S.D, & Keane, S.P. (2004). Cardiac vagal regulation across the preschool period: Stability, continuity, and implications for childhood adjustment. *Developmental Psychobiology, 45,* 101-112.**

Keane, S. P. & Calkins, S.D. (2004). Developmental trajectories of early behavior problems: Implications for kindergarten social status. *Journal of Abnormal Child Psychology, 32,* 409-423.

Keane, S.P., & Calkins, S.D. (2004). Predicting kindergarten peer social status from toddler and preschool problem behavior. *Journal of Abnormal Child Psychology, 32,* 409-423.

Smith, C.L., Calkins, S.D., Keane, S.P., Anastopoulos, A.D., & Shelton, T.L. (2004). Predicting stability and change in toddler behavior problems: Contributions of maternal behavior and child gender. *Developmental Psychology, 40,* 29-42.

Gill, K.L., & Calkins, S.D. (2003). Do aggressive/destructive toddlers lack concern for others? Behavioral and physiological indicators of empathic responding in 2-year-old children. *Development and Psychopathology, 15,* 55-71.

Howse, R.B., Calkins S.D., Anastopoulos, A.D., Keane, S.P., & Shelton, T. L. (2003). Regulatory contributors to children’s kindergarten achievement. *Early Education* *& Development, 14,* 101-119.

Calkins, S. D., & Dedmon, S.E. (2000). Physiological and behavioral regulation in two-year-old children with aggressive/destructive behavior problems. *Journal of Abnormal Child Psychology, 28,* 103-118.

Calkins, S.D., Gill, K.L., & Williford, A.P. (1999). Externalizing problems in two-year-olds: Implications for patterns to social behavior and peers’ responses to aggression. *Early* *Education & Development, 10,* 267-288.
